# Ontogenetic changes in energetic reserves, digestive enzymes, amino acid and energy content of *Lithodes santolla* (Anomura: Lithodidae): Baseline for culture

**DOI:** 10.1371/journal.pone.0232880

**Published:** 2020-05-13

**Authors:** Hernán Javier Sacristán, Jesica Romina Mufari, Rodrigo Antonio Lorenzo, Claudia Clementina Boy, Gustavo Alejandro Lovrich

**Affiliations:** 1 Centro Austral de Investigaciones Científicas (CADIC), CONICET, Ushuaia, Argentina; 2 Instituto de Investigaciones Biológicas y Tecnológicas (IIByT), CONICET, Córdoba, Argentina; Evergreen State College, UNITED STATES

## Abstract

The southern king crab (SKC) *Lithodes santolla* is an important commercial species in southern South America. Fishing pressure has caused the deterioration of its stocks. Currently, culture techniques are being developed for producing SKC juveniles to enhance the natural population and to recover the fishing stock. Therefore, it is necessary to know about physiology, energetic and nutritional requirements for SKC maintenance in hatchery. Thus, this study aims to evaluate the biochemical and physiological changes in the midgut gland, muscle and hemolymph of juveniles, pre-adults and adults of wild SKC. The energetic reserves, digestive enzymes activity, amino acid profile and energy were quantified in twelve juveniles, ten pre-adult, and ten adult crabs. Juveniles showed high glycogen and low lipids in the midgut gland, and low proteins and low lactate in muscle. In the hemolymph, juveniles had high lipids. Pre-adults had high glycogen and lipids in the midgut gland, and both high protein and lactate in muscle. In the hemolymph, pre-adults had high lipids. Adults had low glycogen and high lipids in midgut gland, and both high proteins and high lactate in muscle. In hemolymph, adults had high glucose and lactate. Juveniles and pre-adults had high proteinase activity, whereas adults had high lipase activity. Major essential amino acids of SKC were arginine, methionine, and tryptophan, and the non-essential amino acids were glycine, aspartic acid and glutamic acid. On another hand, SKC had similar energy in the midgut gland and muscle, regardless of the ontogenetic stage. Moreover, we demonstrated that the biochemical energy calculation underestimates the actual measured values by a calorimeter. Thus, our results help to understand the physiological changes, energetic and nutritional requirements of *L*. *santolla*, and this study is a baseline for research on diet formulation for maintaining this species under culture conditions.

## Introduction

The southern king crab (SKC) *Lithodes santolla* (Anomura: Lithodidae) has a high commercial value and has been fished in southern South America since the 70s. In the last five years, landings have averaged ca. 9,000 t per year [1], with most captures from Chilean waters mainly south of 50°S, and the Atlantic waters near the city of Comodoro Rivadavia, Argentina, ca. 45°S 60°W [2,3]. The Argentine fishery for *L*. *santolla* in the Beagle Channel is comparatively smaller, with landings in Argentina and Chile of ca. 50 t and 700 t per year during the last five years respectively [4]. The Argentine fishery for SKC of the Beagle Channel collapsed in 1993 due to two factors: the high fishing pressure over a small area near the city of Ushuaia (ca. 54°S 68°W), and to the lack of controls that permitted illegal landings of females and sublegal male crabs. Thus, the fishery for both commercial species *L*. *santolla* and *Paralomis granulosa* remained closed between 1994 and 2013. Notwithstanding this long closure, recent surveys demonstrate that the population remains vulnerable: e.g., the relative abundance as catch-per-unit-effort is 25% of abundance first recorded in 1975, along with only ca. 30% of ovigerous females [5,6]. Currently, the combined techniques of indoor (hatchery) and outdoor culture (sea culture) are being tested for the production of a large number of SKC juveniles to enhance the natural population and for the fishery stock recovery [7].

In lithodid species, the knowledge about nutritional and energy requirements is scarce, although it is recognized its importance for crab maintenance in cultures. A suboptimal diet can reduce the growth of crabs cultured in the laboratory in relation to wild crabs, and can promote cannibalism [7–9]. Studies on physiologic and energetic changes throughout ontogenetic development of *L*. *santolla* can be useful for understanding their nutritional requirements, for an adequate diet formulation and optimization of the culture practice, and allows understanding the SKC ecological role in the ecosystem.

Development and growth of animals imply conversions of matter and potential chemical energy, either originated from ingested food or mobilized from stored somatic reserves [10]. In this regard, the midgut gland or hepatopancreas of crustaceans plays a key role during growth and molting because it is the principal organ for synthesis and secretion of digestive enzymes, and absorption and storage of nutrients, such as lipids and glycogen [11,12]. Given the relationship between diet and the produced digestive enzymes, the presence and activity of digestive enzymes can be used as indicative of the relative importance of each component in the diet [13–15]. For example, high proteinase activity was observed in lobsters *Jasus edwardsii*, *J*. *lalandii* and *Thenus orientalis* fed with a protein-rich natural diet, composed by bivalves, crabs, ophiuroids and sponges [14]. High lipase activity was found in juveniles and adults *J*. *edwardsii*, suggesting that dietary lipid can be readily hydrolyzed by this lobster [14].

The stored glycogen is mobilized as an adaptation to molting, growth, hypoxia and/or anoxia, osmoregulation, in the different stages of reproduction, and during starvation periods [16–22]. In the hemolymph, glucose comes either directly from the absorption of dietary glucose through midgut gland and intestinal epithelial cells or, from the midgut gland where it is stored as glycogen or synthesized by the gluconeogenic pathway [23]. Moreover, in crustaceans the midgut gland is the main organ of lipid storage in crustaceans [16,17,24–28], although lipids can also accumulate in the muscle and the female gonads [29]. Early developmental stages require high protein levels for fast tissue synthesis [10]. Amino acids, the protein precursors, must be synthesized by mobilizing from the protein storage (non-essential amino acid, NEAA), or ingested through food (essential amino acid, EAA) [10]. The amino acid profile of the body tissue can be used to predict the dietary amino acid requirement [30–32].

Under the aquaculture perspective, an understanding of the energetics of different crustacean stages is an essential issue, since the growth of crustaceans may be related to the survival of individuals during their early stages [33]. Since direct measurements of the energetic content and metabolic heat production are not always possible, the parameters of bioenergetic budgets are usually estimated with indirect methods. The caloric content of the crustacean body mass can be measured directly, using a calorimetric bomb or wet oxidation technique [10]. Alternatively, it may be estimated from the average energetic contents of the major biochemical compound classes as carbohydrates, lipids and proteins.

Thus, the aim of this study was to evaluate the biochemical and physiological changes through the quantification the energetic reserves, digestive enzymes, amino acid profile and energy in the midgut gland, muscle and hemolymph of juveniles, pre-adults and adults of wild *L*. *santolla*, to provide relevant information to improve indoor culture practice. We also provide tissue- and species-specific conversion factors to estimate energy content when direct measurements are not possible.

## Materials and methods

### Animals

Twelve juvenile crabs (six males and six females; averaging 14.40±2.67g body mass; 27.75±2.16 mm carapace length, CL) and ten pre-adult crabs (six males and four females; averaging 160.35±13.32 g body mass; 62.10±1.61 mm CL) of *L*. *santolla* in intermolt stage, were collected by scuba diving at depths of 6–12 m from the subtidal zone of the Golondrina Bay (54° 50´S 68° 21´W) in the Beagle Channel, Ushuaia. Ten adult crabs (five males and five females; averaging 940±125 g body mass; 106.78±4.51 mm CL) were captured in Punta Oriental (54° 50´S 68° 15´W), Beagle Channel using baited commercial traps. This categorization was based on the attaining of gonadal maturity at a size of 70 mm CL or 75 mm for males or females, respectively [34]. The crabs were transported to the aquarium facilities of the Centro Austral de Investigaciones Científicas (CADIC), Ushuaia, Tierra del Fuego, and kept in individual plastic containers with a chilled seawater recirculation system at 6–8°C, a photoperiod cycle of 10 h light:14 h dark (resembling the Beagle Channel day/night cycle at the date of the capture), and without food. Water quality was maintained using mechanical (50 μm) and biological filters, and a UV-sterilizer. Under this condition, the crabs were maintained for 48 h until dissection.

### Sampling collection

Hemolymph samples were collected from the base of the pereopod using a hypodermic syringe (G-20). Hemolymph of each crab was carefully withdrawn and kept independently in microtubes (1.5 mL) with precooled anticoagulant solution (10mM HEPES. 1:2 v/v). After centrifugation at 800 x g for 3 min at 4°C, the supernatants were rapidly frozen (-80°C) and stored for further biochemical analysis. Later, all crabs were anesthetized, measured, and weighed. Each midgut gland and pereopod muscle was dissected and frozen at -80°C.

### Energetic reserves of the midgut gland and muscle

Glycogen concentration was determined in small samples (30–50 mg) of the midgut gland and muscle through basic digestion (30% KOH saturated with Na_2_SO_4_) and precipitated with ethanol 96° following Lo et al. [35]. Samples were placed on ice for 30 min and then were centrifuged at 4,500 rpm for 10 min. The glycogen precipitates were next dissolved in 1 mL of distilled water. An aliquot of 300 μL of the above glycogen solution was brought to a sample volume of 1 mL by the addition of distilled water, 0.5 mL of 8% phenol solution, and 2.5 mL of H_2_SO_4_. Subsequently, the tubes were allowed to stand for 10 min, shaken and placed for 20 min in a water bath at 25–30°C, and readings were taken. The absorption spectrum was read at 490 nm, and the standard solution was prepared with rabbit glycogen (Sigma G0885). Glycogen content was expressed as milligram of glycogen per gram of tissue.

Total lipids were extracted by homogenizing pre-weighed samples of the midgut gland or muscle, and hemolymph with chloroform:methanol (2:1 v/v), and the homogenate was filtered through a funnel with a filter paper to recover the liquid phase following Folch´s protocol [36]. Total lipids were determined by the sulfophospho-vanillin method [37]. This method consists of oxidizing cellular lipids to small fragments after chemical digestion with hot concentrated sulfuric acid. After the addition of a solution of vanillin and phosphoric acid, a fuchsia complex was formed and its absorbance was read at 530 nm on a CINTRA 10e GBC spectrophotometer. The standard solution was prepared with commercial extra virgin olive oil (Cocinero, Molinos Río de la Plata S.A., Argentina). Lipid concentration was expressed as milligrams of lipids per gram of tissue.

Total soluble protein from midgut gland, muscle and hemolymph were evaluated with the Coomassie blue dye method using serum bovine albumin as the standard (Sigma A6003) [38]. Soluble protein content was expressed as milligram of protein per milliliter of supernatant.

Finally, hemolymph glucose and L-Lactate of the midgut gland, muscle and hemolymph were evaluated according to Ridgway et al. protocol [39] with minor modifications. Briefly, deproteinization was achieved as follows: homogenization of samples (3:1 v/v) with 75 μL perchloric acid (2 M) and further centrifugation for 10 min at 10000 g and 4°C, neutralizing supernatants with 75 μL KOH (2 M). The resulting KClO_4_ salts were removed by 2 min of centrifugation at 10,000 g and 4°C; later the precipitate was discarded. Glucose and L-Lactate were measured using a commercial kit (Weiner lab, Rosario, Argentina).

### Amino acid profile of muscle

The muscle samples were subjected to acid hydrolysis with 6N HCl under reflux for 24 h following AOAC 994.12 methodology [40]. Amino acids identification and quantification were evaluated using a high-performance liquid chromatographer (HPLC), with UV-detector (Perkin Elmer 600 Series, United States), and data acquisition and processing were done using a Total Chrom Workstation software (version 6.3). The separation was performed with a Zorbax Eclipse Plus C18 column (4.6 × 150 mm and particle size of 5 μm) Agilent Technologies, with previous derivatization of the amino acids with diethyl ethoxymethylenemalonate [41]. Following, identification of sample compounds was done by using a standard external method, comparing the obtained chromatogram with an amino acid standard (AAS18, Fluka Analytical, Sigma Aldrich). For quantification, calibration curves for each amino acid were constructed from the standard solution (with adjustment coefficients between 0.992 and 1). Tryptophan amino acid was determined in muscle samples according to the protocol of Yust et al. [42] through basic digestion at 100°C for 8 h, neutralization of the resulting hydrolysate to pH 7, dilution with sodium borate buffer (pH 9), and analysis by reverse-phase high-performance liquid chromatography with spectrophotometric determination of tryptophan at 280 nm. The samples were hydrolyzed in duplicate, and each duplicate was injected three times. The mean value of the six determinations was reported as mg of amino acids per 100 g of protein.

### Activity of digestive enzymes in the midgut gland

Each midgut gland (100–150 mg) was homogenized in cold Tris-HCl (50 mM, pH 7.5, 1:4 w/v) in an ice-water bath using a potter homogenizer. After centrifugation (Sigma 3-18K) at 10,000 x *g* for 30 min at 4°C, the lipid layer fraction was removed, and the supernatant (enzyme extract) was stored at -80°C until used as enzyme extract.

Total proteinase activity was assayed using 1% azocasein as the substrate in 50 mM Tris HCl, pH 7.5 [43]. Absorbance was recorded at 440 nm.

The lipase activity of each enzyme extract was determined according to Versaw et al. [44]. The assay mixture consisted of sodium taurocholate (100 mM), buffer TRIS-HCl (50 mM, pH 7.5) and the enzyme extract. After incubation (25°C for 5 min), the substrate β-naphthylcaprylate (Goldbio N-100) dissolved in dimethyl sulfoxide was added to the mixture. This mixture was incubated at 25°C for an additional 30 min before adding 20 μL Fast Blue BB. The reaction was stopped with trichloroacetic acid and clarified with ethyl acetate:ethanol. Absorbance was recorded at 550 nm. One proteinase or lipase unit was defined as the amount of enzyme required to increase the optical density by 0.01 OD units at 440 nm [45].

### Calorimetric determinations

The midgut gland and muscle dry mass of each crab were determined after desiccating at 60°C to constant mass. All dry samples were stored in sealed bags at -20°C until processing. Dry samples for calorimetric determinations were grounded, and pellets were made with a press Parr 2812. The caloric content of each sample was obtained by burning pellets of 20–200 mg in a micro-bomb calorimeter Parr 1425 following Lucas [46] and Boy et al. [47]. The obtained values were corrected for ash and acid content and expressed as J/g tissue AFDW (energy density). Benzoic acid calibrations were carried out periodically.

Alternatively, the biochemical energy of each midgut gland and muscle samples were estimated from the biochemical composition using the following caloric equivalent: 17.15 J/mg for carbohydrates, 39.54 J/mg for lipids, and 23.64 J/mg for proteins [48]. In addition, a conversion factor was calculated between the direct measured energy (calorimetric method; energy density) and the biochemical energy as energy density/biochemical energy.

### Statistical analysis

Data are presented as mean ± standard error. The statistical analyses were performed to compare the ontogenetic changes of SKC in the midgut gland or tissue (muscle or hemolymph) for each energetic reserve, digestive enzyme, lactate, amino acid and energy. The objective variable was the different ontogenetic stages (juvenile, pre-adult, and adult), while the explanation variables were each energetic reserve, digestive enzyme, lactate, amino acid and energy. The ontogenetic stage was treated as a categorical variable. Additionally, comparisons were done between male and female within each ontogenetic stage. Data from biochemical analyses were analyzed using R for Generalized Linear Mixed Models (GLMMs) estimation through the generalized least squares function (gls) and linear mixed effects (lme) procedures of the nlme package using Info-Stat software (2015) [49, 50,51]. The distribution family we used was Normal. The heterogeneous variance structure was modeled, and the most parsimonious model was selected using the Akaike Information Criterion (AIC), and graphical inspection of residual distributions. Post-hoc comparisons were performed using Fisher's LSD test. For all analyses, residuals were analyzed for normality using the Shapiro-Wilks test.

## Results

We observed no significant differences in all biochemical analyses performed between male and female SKC (p<0.05). Therefore, we pooled data within each of the three ontogenetic stages: juveniles, pre-adults and adults.

**Fig 1:**
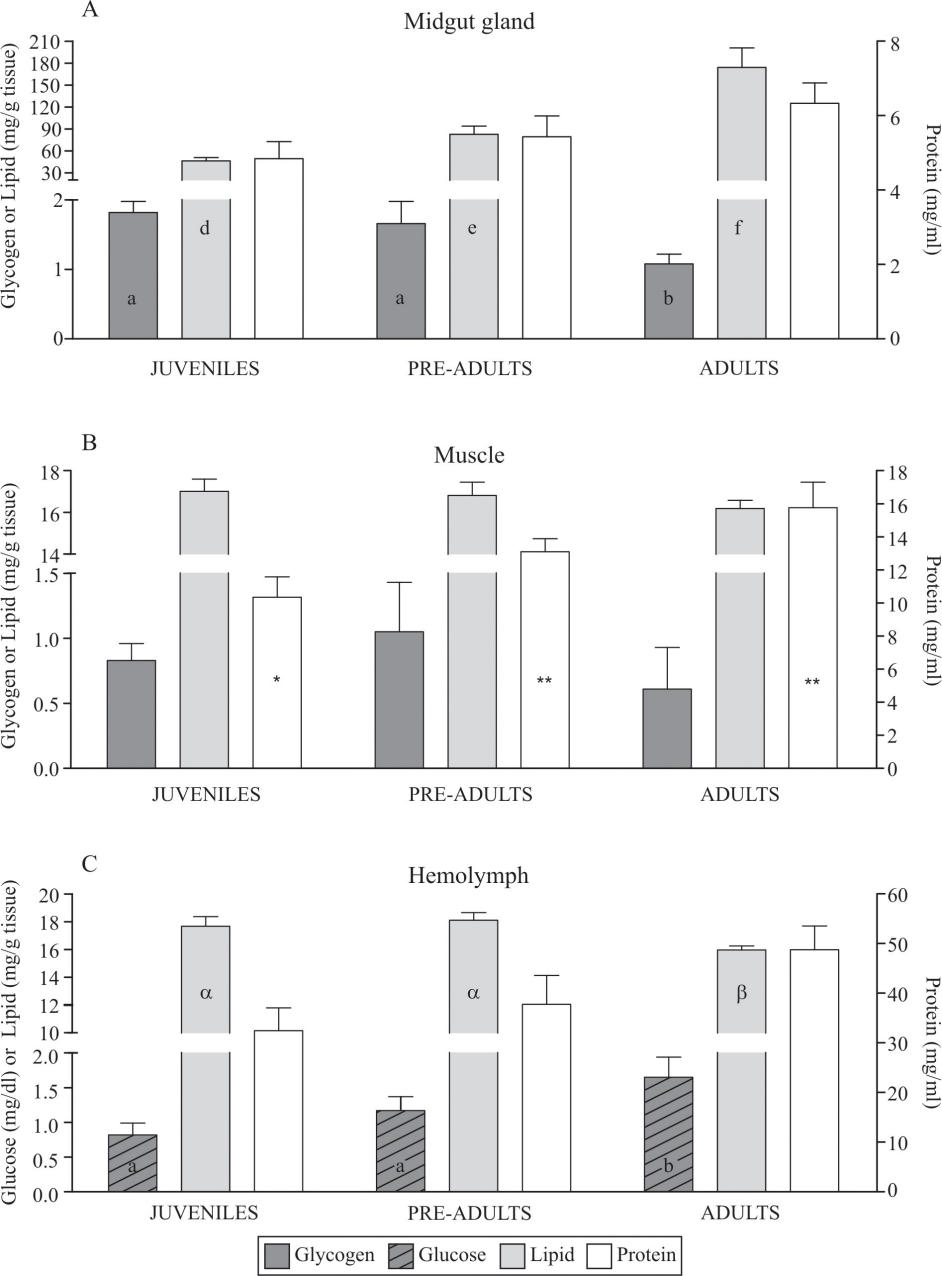
Glycogen, glucose, lipid, and protein concentrations of the midgut gland (A), muscle (B) and hemolymph (C) of *Lithodes santolla* at different ontogenetic stages. Different letters (a, b or α, β, χ) and asterisks (single or double) indicate statistical differences among ontogenetic stages (p<0.05).

In the midgut gland of *L*. *santolla*, the glycogen reserves were higher in juveniles and pre-adults than in adults. Conversely, lipids levels increased ontogenetically, with the lowest in juveniles and the highest in adults (p = 0.04). The soluble protein of the midgut gland remained unchanged among the three ontogenetic stages (p = 0.17) (Fig 1A). In contrast, in the muscle, pre-adults and adults had more soluble protein than in juveniles (p = 0.01) (Fig 1B). The glycogen and lipids of the muscle remained unchanged (p = 0.67 and p = 0.44, respectively) (Fig 1B). In the hemolymph, adults had higher glucose (p<0.05) and lower lipid concentrations (p<0.05) than in juveniles and pre-adults. Protein levels of the hemolymph of SKC remained unchanged during the ontogeny (p = 0.07) (Fig 1C).

**Fig 2:**
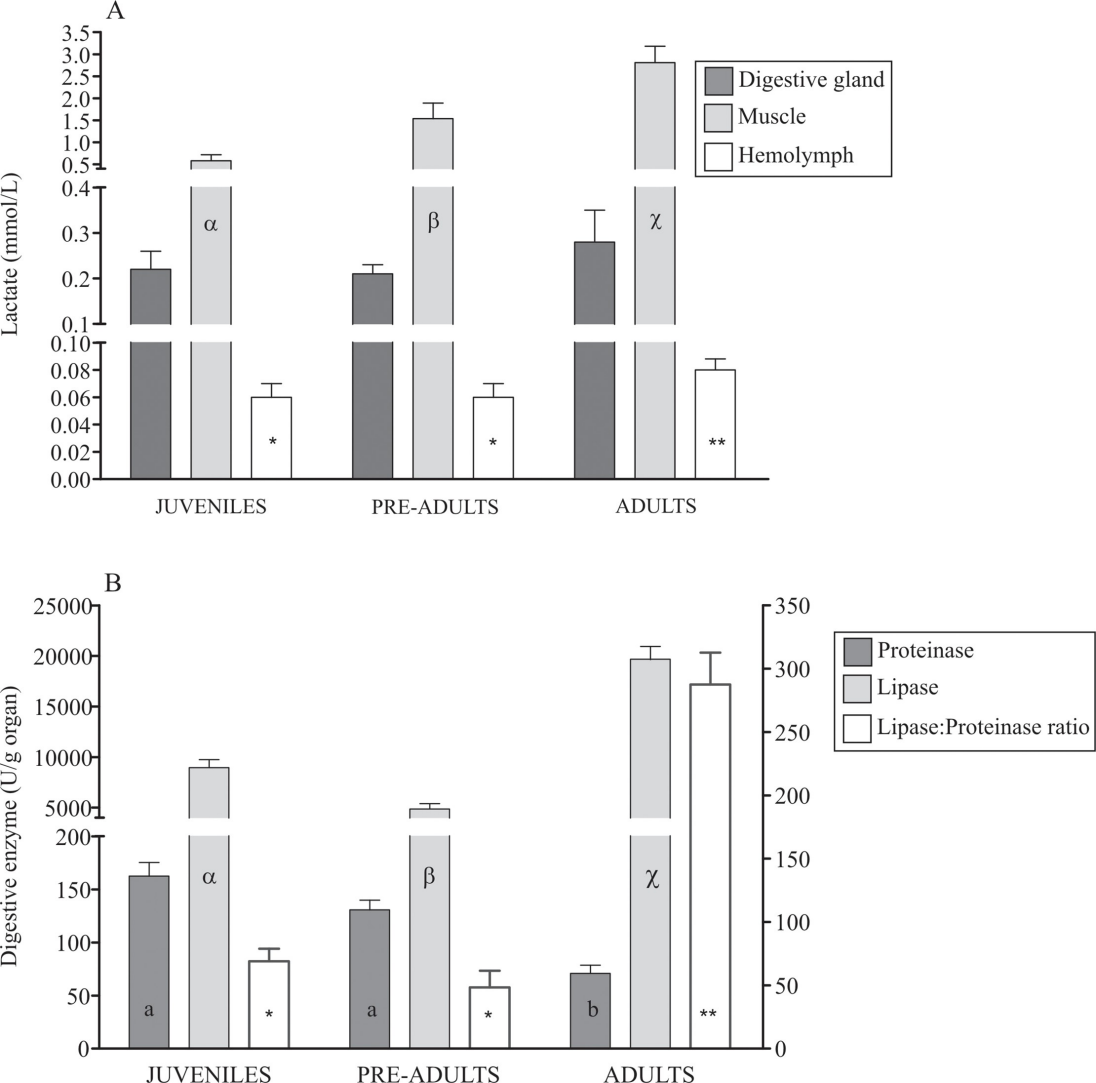
Lactate concentrations of the midgut gland, muscle and hemolymph (A); activity of digestive enzyme and lipase:proteinase ratio in the midgut gland (B) of *Lithodes santolla* at different ontogenetic stages. Different letters (a, b or α, β, χ) and asterisks (single or double) indicate statistical differences among ontogenetic stages (p<0.05).

In the muscle, lactate levels increased with crab size (p<0.05), but in the midgut gland, the lactate level was similar (p = 0.61) (Fig 2A). Adult *L*. *santolla* had more lactate in the hemolymph than juveniles and pre-adults (p = 0.02) (Fig 2A). In contrast, juveniles and pre-adults had higher proteinase activity than adults (p<0.05) (Fig 2B). However, adults had the highest lipase activity, followed by a significant decreasing lipase activity in juveniles and pre-adults (p<0.05) (Fig 2B). Adult *L*. *santolla* had high lipase:proteinase ratio than juveniles and pre-adults (p<0.05) (Fig 2B).

Major essential amino acids of *L*. *santolla* were arginine, methionine, phenylalanine, leucine and tryptophan, and the non-essential amino acids were glycine, aspartic acid, and glutamic acid (p<0.05) (Table 1). Juvenile crabs had higher methionine, tryptophan and cysteine (NEAA) concentrations than pre-adult and adult crabs (p<0.05). However, proline was the lowest in juvenile crabs (p<0.05). Concentrations of total amino acids, EAA, and NEAA were similar ontogenetically (p = 0.49 and p = 0.47, respectively) (Table 1).

**Table 1:** Average (± SE) amino acid composition of the muscle of *Lithodes santolla* at different ontogenetic stages.

Amino Acids (mg/100g dry mass)	Juveniles	Pre-Adults	Adults
*Essential (EAA)*			
Arg	1,041.1±295.5 (11.4%)	1,140.2±229.8 (13.3%)	1,534.1±183.6 (13.9%)
Val	148.3±42.6 (1.6%)	323.2±24.6 (3.8%)	384.9±125.5 (3.5%)
Met	827.3±51.9 (9%)^a^	252.8±70.9 (3%)^b^	311.3±55.5 (2.8%)^b^
Thr	276.6±26.5 (3%)	282.1±101.9 (3.3%)	376.7±56.9 (3.4%)
His	359.4±121.5 (3.9%)	281.4±52.2 (3.3%)	588.4±31.9 (5.3%)
Ile	362.1±41.9 (4%)	362.6±70.1 (4.2%)	446.3±66.4 (4%)
Leu	526.8±60.4 (5.8%)	544.1±95.6 (6.4%)	638.1±90.7 (5.8%)
Phen	658.1±65.3 (7.2%)	687.4±110.7 (8%)	764.8±90.1 (6.9%)
Lys	55.17±11.6 (0.6%)	29.85±3.8 (0.3%)	102.62±26.4 (0.9%)
Trp	1,156.25±19.09 (12.6%)^a^	878.25±25.65 (10.3%)^b^	723.50±8.63 (6.6%)^c^
Total EAA	5,411±207.95	4,782±314	5,871±280
*Non-essential (NEAA)*			
Asp + Asn	614.1±154.7 (6.7%)	645.7±192.1 (7.5%)	694.1±58.2 (6.3%)
Glu + Gln	723.1±109.2 (7.9%)	560.4±90.1 (6.6%)	1,061.2±165.4 (9.6%)
Ser	268.3±63.1 (2.9%)	186.6±120.1 (2.2%)	340.7±53.9 (3.1%)
Gly	1,014.9±295.5 (11.1%)	1,263.8±41.5 (14.8%)	1,772.4±210.3 (16.1%)
Ala	238.3±20.3 (2.6%)	221.1±36.9 (2.6%)	266.7±43.5 (2.4%)
Pro	42.5±17.6 (0.5%)^a^	208.6±43.2 (2.4%)^b^	261.1±51.9 (2.4%) ^b^
Tyr	617.6±70.8 (6.8%)	630.4±98.6 (7.4%)	274.9±79.1 (6.6%)
Cys	213.1±21.7 (2.3%)^a^	56.1±29.1 (0.7%)^b^	87.1±16.7 (0.8%)^b^
Total NEAA	3,732±365	3,772±273	5,158±298
Total Amino Acids	9,144±420	8,554±2,320	11,029±3,124

Numbers in parentheses indicate the percentage of each amino acid relative to the total number of amino acids. For each amino acid, different letters indicate statistical ontogenetic differences (p<0.05).

*Lithodes santolla* had a similar energetic density in the midgut gland and muscle (p = 0.08 and p = 0.19, respectively), regardless of the ontogenetic stage (Table 2). However, in the midgut gland, the biochemical energy increased with crab size (p<0.05) but remained unchanged in the muscle (p>0.05). The conversion factor between calorimetric and biochemical methods of the midgut gland was higher in juveniles (2.71±0.16) than in pre-adults (2.17±0.07) and adults (1.80±0.27) (p = 0.01). In the muscle, the conversion factor was similar between ontogenetic stages (p = 0.58) (Table 2).

**Table 2:** Energy density, biochemical energy and conversion factor between calorimetric and biochemical methods in the midgut gland and muscle of *Lithodes santolla* at different ontogenetic stages.

		Juveniles	Pre-Adults	Adults
Midgut Gland	Energy density (J/g tissue AFDW)	14,990±1150	17,460±860	18,630±1000
	Biochemical energy (J/g tissue)	5,879±625^a^	7,942±401^b^	10,792±1192^c^
	Conversion factor	2.71±0.16^a^	2.17±0.07^b^	1.80±0.27^b^
Muscle	Energy density (J/g tissue AFDW)	13,980±480	14,840±170	15,180±480
	Biochemical energy (J/g tissue)	8,154 ± 513	8,956 ± 754	9,254 ± 1205
	Conversion factor	1.75±0.15	1.51±0.18	1.78±0.22

Different letters indicate statistical ontogenetic differences (p<0.05)

## Discussion

The present study provides new and relevant biological information on physiological changes during ontogeny of *L*. *santolla* that can improve its culture. The main results of our research reveal that the SKC juveniles, pre-adults and adults present dissimilar physiological profiles according to multiple parameters as glycogen and lipids reserves, lactate, proteinase and lipase enzymes, amino acids profile and energy content.

According to our results, lipids are the most important energetic reserve in adult crabs, probably associated with reproduction. Contrarily, glycogen is the most important energetic reserve during the first years of crabs, perhaps associated with high both metabolism and molting rate. Specifically, the lipids reserves increase ontogenetically, whereas the glycogen reserves of the midgut gland decrease in adult crabs. Molt frequency progressively decreases with crab size. *Lithodes santolla* adults molt annually, whereas juveniles molt at least 20 times during the first three years of their life span [3]. In crustaceans, ecdysis is a process that requires high levels of energy [10]. The high molting frequency of the juvenile crabs coincides with their high metabolism demand and thus the storage of glycogen as a molecule of rapid availability of energy. On another hand, the higher lipid concentration in adults is related to the reproductive and growing processes. In *Lithodes santolla* the oogenesis last approximately two years, with the primary–protein/vitellogenesis during the first year, and the secondary vitellogenesis -lipidic- in the following year [52]. Females undergo secondary vitellogenesis, i.e., accumulation of lipo-proteins in the oocytes, all years between April and December. Coincident with the ovary ripeness, mating female molting, and extrusion of mature oocytes for their fertilization occur simultaneously [34,53]. These energetic demanding processes should be provided with molecules that can be stored for long periods, like lipids, either in the midgut gland or in the ovary, depending on their destination, either molting or reproduction. There are few studies about energetic reserves of male crabs associated with growth or reproduction. Adult male *L*. *santolla* shows maximum lipid levels in the midgut gland before the molting period (mainly in May). However, lipid reserves in their midgut glands remained unchanged with mating, although males likely starve during this period [53,54].

During the ontogeny of *L*. *santolla*, the capability to digest lipids was higher than for proteins, and it changes with size. Juvenile and pre-adult crabs had higher protein digestive capacity, while adult crabs had higher lipid digestive capacity (Fig 2B). In this study, we attribute the high activity of one specific digestive enzyme to the utilization of a particular biochemical component of food. For example, carnivorous crab *Plagusia chabrus* has high protease and trypsin activities to break down the high proteic diet [55]. Therefore, our results suggest that juveniles and pre-adults crabs feed on protein-rich foods, while adult crabs feed on lipid-rich foods. In SKC food habits change ontogenetically [56]. Specifically, in the Beagle Channel, crabs <50 mm LC feed on gastropod mollusks; crustaceans such as anphipods, copepods and isopods; bryozoans; echinoderms, and foraminiferans, as an indirect evidence of foraging on macroalgae. Crabs between 50–70 mm LC prey on mollusks; crustaceans such as isopods and decapods; while adult crabs between 90–100 mm LC mainly consumes gastropod mollusks, decapods crustaceans and echinoderms. Small crabs generally ingest more food than larger crabs [56–59] and the prey size was directly proportional to crab size [60]. This was hypothesized as a larger energy demand during growth, due to a higher molting frenquency and percent increment per molt among smaller crabs [56].

The requirements of dietary protein are particularly high in rapidly growing early life-history stages, with a high metabolism, to provide tissue synthesis [10]. Besides, the proteinase activity corresponds with the natural food sources of *L*. *santolla* juveniles [61–64], and with the high proteinase activity already observed in the first crab stage (C1), which contributes to the extracellular digestion at the start of feeding [65]. On the other hand, our hypothesis that adult crabs feed on lipid-rich foods is coincident with the high lipid reserve concentration observed in the midgut gland. Lipids stored in the midgut gland are transferred to the ovary during the vitellogenesis [66]. The polar lipids (phospholipids) are mainly responsible for the increase of lipid concentration of the ovaries during primary vitellogenesis [67,68], while the triglycerides increase during secondary vitellogenesis [68]. The profile of fatty acids present in the maturing ovaries is a reflection of the fatty acid requirement of that tissue or the requirements for the developing embryos after fertilization [67]. In crustaceans with lecithotrophic larvae, one of the most important roles of lipids is related to reproduction, since they are associated with the maturation of oocytes and the survival of the whole larval period [69,70 and references therein]. Lipids of the eggs are key macromolecules in crustacean species with lecithotrophic larval development such as *L*. *santolla*, *Paralomis granulosa* [71], and *Homarus gammarus* [72]. Lipids will constitute the energetic reserves in larvae to survive all the larval period that lasts 60 days [10]. Since maternal effect is strong on fully lecitotrophic larvae (e.g. [73]; [69]), the nutritional condition of mothers during the secondary vitelogenesis is crucial for the larval quality [74–76]. We therefore hypothesize that female nutritional condition can be used as a proxy for selection of better animals for culture and enhancement of the stock of *L*. *santolla*.

The lactate level reflects protein and lipid catabolism. Lactate increases gradually with crab size in the muscle (Fig 2A), and is coincident with the high protein concentration observed in pre-adult and adult SKC (Fig 1B). Moreover, adult crabs showed high lactate and glucose levels in the hemolymph. Several studies have demonstrated that the gills [77], muscle [78,79], and hemocytes [80] are sites of glucose synthesis in crustaceans. In the crab *Nehoelice granulata*, gluconeogenesis from lactate occurs in the midgut gland [81]. However, the muscle can also due have gluconeogenic potential form lactate [78,81–83]. Therefore, from our results, the glucogenic conversion of the protein and/or fatty acids to glucose synthesis from lactate, lipids (by Citric Acid Cycle), and amino acids (such as alanine, serine, glycine, others) appears to be a mechanism for increasing hemolymph glucose and maintaining muscular glycogen levels.

The increase of L-lactate in *L*. *santolla* during ontogeny could be due to an increase in the amount of lactate dehydrogenase activity, the locomotion activity and/or the hemolymph volume. In the muscle, lactate increases with the ontogenetic stage of *L*. *santolla*. In the hemolymph, adult crabs had higher lactate than juvenile and pre-adult crabs. The glycolytic enzyme lactate dehydrogenase (LDH) catalyzes the conversion of pyruvate to lactate. In snow crab, *Chionoecetes opilio*, there are different LDH expression pattern between immature and mature male, where LDH expression increase in mature animals [84]. *Jasus edwardsii* pueruli presents high LDH activity, when compared with its successive stage, phyllosome [85]. Moreover, after intense exercise lactate accumulates in hemolymph, as in the crab *Gecarcoidea natalis* [86]. Therefore, an increase in locomotion could be related to higher lactate basal concentrations and thus explain the increased concentration in adults. In addition, extensive extracellular fluid is less vulnerable to acidification, and thus greater hemolymph volume could buffer higher lactic acid levels [87]. In addition, total hemolymph volume is related to the crab body weight [88], which implies bigger animals and thus longer distances for tissue oxygenation. Moreover, the binding of lactate to hemocyanin increases oxygen affinity [89], without affecting the respiratory pigment active site [90], a useful effect to cope with longer oxygen-perfusion distances. Finally, several factors can explain the increase in lactate concentration throughout ontogeny, but further studies are needed to understand this topic in SKC ontogeny.

In studies on amino acid requirements, the reference of the dietary amino acid profile is mostly based on either crustacean whole body or tail muscle composition [91,92]. Our information allows to obtain the essential amino acid requirements of the species, the possibility to formulate diets, and the feasibility of incorporating vegetable protein sources to diets. According to our results, a meal with high amounts of methionine, arginine, tryptophan and cysteine amino acids will be needed for an adequate diet formulation in order to maintain *L*. *santolla* juveniles in culture. Muscle of *L*. *santolla* juveniles has high amounts of methionine, arginine, tryptophan (9%, 11.4% and 12.6% respectively) than the muscle of pre-adults and adults. In industrially manufactured shrimp feeds, methionine is considered the most limiting essential amino acid [93]. Methionine and arginine are essential amino acids that are critical for optimal growth and survival of crustaceans [94–97]. Also, *L*. *santolla* has high concentrations of NEAA: aspartic acid, glutamic acid and glycine (6.8%, 8% and 14% respectively). Similar results were found in other decapod crustaceans. The wild crab *Cancer pagurus* has the same three NEAA that *L*. *santolla* (glutamic acid, aspartic acid and glycine), and as for EAA: arginine, leucine and threonine [98]. The cultured shrimp *Penaeus vannamei* juveniles present high levels of the glycine, arginine, proline and alanine [99]. In immature and mature wild shrimps *Aristeus antennatus* and *Parapenaeus longirostris*, and the wild lobster *Nephrops norvegicus* the major EAA are arginine, lysine and leucine, and the quantitatively most important NEAA are glutamic acid, aspartic acid, proline and glycine [100]. Finally, in both shrimp and lobster species, the limiting amino acid is methionine [101]. Variations in amino acids that cannot be synthesized *de novo* (EAA) reflect the dietary condition [10], and deficiencies or excesses of one or more EAA limit protein synthesis, growth or both [102–104]. Amino acid deficiencies provoke low survival of *Pacifastacus leniusculus* juveniles after 100 days of culture [105]. Our work is the first reporting an amino acid profile of the muscle of *L*. *santolla*. Thus, the information attained here allows to obtain the essential amino acid requirements of this species and also gives the possibility to formulate a diet for SKC growth improvement, a key feature in juvenile crabs culture.

The knowledge about the amino acid profile of juvenile crabs could have a direct application by reducing cannibalism in cultures. For example, the essential amino acid tryptophan is the precursor of serotonin (5-hydroxytryptamine), a neurotransmitter that is linked to agonistic behavior [106]. Also, dietary supplementation with high tryptophan levels causes an increase of serotonin concentration, and thus suppressing aggression and improving the survival of some cultured fish [107–109] and decapod crustaceans [110]. In this study, we observed that the muscle of *L*. *santolla* juveniles has high amounts of tryptophan. However, under laboratory conditions, cannibalism is frequent when SKC´s early stages are pooled together, and this behavior occurs during both intermolt and molting periods [7]. Cannibalism is one of the main reasons for failures in the development of culturing methods for a variety of crab species [7,111,112]. It can be triggered to compensate for nutritional deficiencies and/or low feed palatability [113]. During the early stages of crustacean juveniles, cannibalism can be particularly intense, because of their high metabolic and growth rate [113], which, in turn, causes losses in cultures through mortality.

Throughout its ontogeny, *L*. *santolla* exhibits similar energy density in the midgut gland and muscle. In contrast, the variation of biochemical composition in ontogeny of SKC is reflected in the profile of biochemical energy. The biochemical energy of the digestive gland increased with crab size, whereas it was constant in the muscle. Specifically, in the midgut gland, the biochemical energy follows the pattern of the lipids concentration, the macromolecule of greatest concentration (Fig 1A). Our results demonstrate that the estimation of biochemical energy underestimates the measured values as the calorimetric method. Similar differences between both methods were observed in *Penaeus paulensis* [114]. One possible explanation is that biochemical determinations quantify the total lipids, regardless of different lipids class (triacylglycerides, free fatty acids, wax esters, sterols), which have different energetic contents. Soluble proteins were measured through the Bradford method, by which the integral membrane proteins are discarded because they remain in the pellet after homogenization and centrifugation. Lemos and Phan [114] explain that the protein reagent Coomasie Brilliant Blue is known to bind mainly on hydrophobic amino acids. Glycine is one of the quantitatively most important non-hydrophobic amino acids of decapod crustaceans including *L*. *santolla* [present study; [115,116]. Consequently, when protein is the most abundant component, any underestimation of protein levels would lead to considerably lower energy estimated values [114].

When calorimetric measurements are not possible to perform, we propose the use of conversion factors to estimate the energy from the biochemical composition of *L*. *santolla*. Factors are for muscle 1.68, and two stage-specific conversion factors for midgut gland: 2.71 for juvenile crabs and an average of 1.99 for pre-adult and adult crabs. Probably, the conversion factor is species-specific, varies with sexual maturity, and could be different for the same species of different regions due to differential feeding habits.

The experimental data obtained may have practical utility in understanding the body nutrient levels of *L*. *santolla* and the amino acid profile of the body tissues. The biochemical and nutritional data of the *L*. *santolla* given above might help understand the dietary requirements of SKC. This, in turn, will help in formulating the balanced diets according to the nutritional requirements of the SKC.

Finally, according to physiological changes observed through *L*. *santolla* ontogeny, we conclude that juvenile crabs have high protein dietary requirements, and with methionine, arginine, tryptophan, and cysteine as crucial amino acids during the early development. Pre-adult crabs present similar dietary requirements than juveniles, although they have a comparable amino acid profile than adult crabs. Based on previous studies and our present results (lipid digestive capacity and lipid reserve of the midgut gland), we propose that adult crabs obtain the energy mainly from dietary lipid. Thus, our results help to understand the physiology, energetic and nutritional requirements of SKC *L*. *santolla*, and this work is the starting point for current research on diet formulation for maintaining this species of high commercial value in culture condition.
