# Supplementary material for: Ontogenetic changes in energetic reserves, digestive enzymes, amino acid and energy content of Lithodes santolla (Anomura: Lithodidae): Baseline for culture
Source: PLoS One. 2020 May 13;15(5):e0232880. doi: 10.1371/journal.pone.0232880 (PMC7219783; doi:10.1371/journal.pone.0232880)
Supplement: Attachment — Submitted filename: Response to Reviewers.docx [file pone.0232880.s001.docx]

April 2020

Dr. Erik V. Thuesen

Academic Editor – PLOS ONE

Dear Dr. Thuesen

We originally submitted the manuscript entitled “Ontogenetic changes in energetic reserves, digestive enzymes, amino acid and energy content of *Lithodes santolla* (Anomura: Lithodidae): Baseline for culture” and we were asked to consider the reviewers comments carefully on December 20, 2019 (PONE-D-19-28115). We really appreciate the constructive criticism to the manuscript by the referees, and we fully agree with their comments. Thus, following the suggestion of resubmitting this manuscript after addressing the concerns expressed by the reviewers, we are sending the revised version.

The following modifications were made to the manuscript in response to the valuable recommendations suggested. All of the points raised by the referees have been modified, and we hope that this version is now acceptable for publication in Plos One.

Summary of changes made to the manuscript.

**Editor comments:**

**I also have some comments for minor corrections.**

**1) Line 275. I don’t understand “(c.a. 1.68)”. Do you mean ‘ca.’ = circa? That is only used for dates. I suggest just deleting “(c.a. 1.68)” or give the overall mean and S.D. for the combined data.**

The c.a. 1.68 was eliminated. Page 13, Line 280

**2) Line 277. Fix Table number to avoid confusion.**

Thank you for this comment. Table number was fixed. Page 13, Line 282

**3) Line 392 should read ‘decapod crustaceans’.**

The word “decapods crustaceans” was changed by “decapod crustaceans”. Page 18, Line 417

**4) Please ignore the first comment of Reviewer #2. Your use of generic abbreviations (or not) is correct in all those spots.**

Ok. I have ignored the first comment of the Reviewer **#**2

**5) Reference problems:**

**#4 Correct authors’ names needed.**

**#15 Italics needed.**

**#73 Fix CO2.**

The references problems were corrected.

**Reviewer #1: This manuscript presents very interesting data on changes in energetic reserves, digestive enzymes activity, amino acid profile and energy with the growth in the southern king crab. The results are clearly presented and this study would provide an important contribution to culturing not only southern king crab but for other king crab species. I recommend this manuscript is acceptable after minor revision.**

**Here are my minor comments.
Statistical analysis.**
**Please clearly write what are the objective variable, explanation variable, random factor, and family in GLMM analyses. Additionally, please add whether the development stage was treated as a categorical variable (juvenile, pre-adult, and adult) or numerical variable (carapace length). In the result section, the result of the GLMM analyses should be shown as a table.**
The objective variable, explanation variable, and family in GLMM analyse, were added in the statistical analysis section. The experimental design does not have a random factor. Page 9; Line 217.

After considering the inclusion of a new table with all the statistical analysis values (see the table below), we decided not to include this new table since it includes too many values that make the table too big. Instead, this results can be describe in the main text, so we included the p-values in the description of the results, since we think this is the most parsimonious option.

|  | | Energetic Reserve | | | | | | |  | |  | | Enzymes | | | |  | |
| --- | --- | --- | --- | --- | --- | --- | --- | --- | --- | --- | --- | --- | --- | --- | --- | --- | --- | --- |
|  | | GLYCOGEN | | LIPID | | PROTEIN | | | GLUCOSE | | LACTATE | | PROTEINASE | | LIPASE | | LIPASE/PROTEINASE RATIO | |
| MIDGUT GLAND | | p=0.04 | | p=0.04 | | p=0.17 | | | ------ | | p=0.61 | | p<0.05 | | p<0.05 | | p<0.05 | |
| MUSCLE | | p=0.67 | | p=0.44 | | p=0.01 | | | ------- | | p<0.05 | | ------- | | ------- | | ------- | |
| HEMOLYMPH | | ------ | | p<0.05 | | p=0.07 | | | p<0.05 | | p=0.02 | | -------- | | -------- | | -------- | |
|  | Amino acids | | | | | | | | | | |  | |  | |  | |  |
|  | MET | | TRP | | PRO | | CYS | EAA | | NEAA | | energetic density | | biochemical energy | | conversion factor | |  |
| MIDGUT GLAND | ------- | | ------- | | ------- | | ------- | ------- | | ------- | | p=0.08 | | p<0.05 | | p=0,01 | |  |
| MUSCLE | p=0.0059 | | p=0.0063 | | p=0.0033 | | p=0.0019 | p=0.49 | | p=0.47 | | p=0.19 | | p>0.05 | | p=0,58 | |  |
| HEMOLYMPH | -------- | | -------- | | -------- | | -------- | -------- | | -------- | | -------- | | -------- | | -------- | |  |

**The authors suggest that juvenile and pre-adult feed on protein-rich foods, while adult feeds on lipid-rich foods. If there has been any previous study on food habits change with growth in the southern king crab or other king crab species, which should be cited in the discussion section.**
This information was added in discussion section. Page 15, Line 325

**Reviewer #2: The study investigated the biochemical and physiological changes of juveniles, preadult and adult of L. santolla. In this regard, the authors make a clear and well-structured approach. The results themselves are interesting but predictable.
The scientific name of the species should be consistent across the manuscript (Line 103, 132, 238, 251, 270, 294….**
**Line 110 and Line 121-122: The juvenile crabs and pre-adult crab were collected at the intermolt stage, but then kept for 48h with feeding or no feeding? Because nutrient availability in the intestine will be effected on the digestive enzyme activities.
Moreover, crabs at intermolt stage were kept for 48h, then what is the real stage of crab? Postmolt? The biochemical compositions and physiological response will be significantly different at pre-molt, inter-molt and post-molt.**

Thank you for both comments. The crabs were kept for 48 h without food. This information was added. Page 5, Line 120.

We agree with the fact that the moulting stage possibly modifies the samples parameters. We are confident that all crabs were all in intermoult stage. The selected size (CL) of crabs molt once, twice, or three times a year (adult, pre-adult and juvenile crabs, respectively) and the time of capture or collect did not match with the moulting period. Previous studies demonstrated that the juvenile crabs of our work (27.8 mm CL) probably during the 3th or 3.5th year molt twice or three times a year. At 62.1 mm CL (CL mean of pre-adult crabs of this work) -probably during the 5th year- females begin to molt annually, since they must derive enough energy for the development of their ovaries (Vinuesa et al., 1991). Gonadal maturity is attained between 65 and 75 mm CL (Vinuesa, 1984) at ca. 5–6 years old, and, thereafter, females molt annually. *Lithodes santolla* males attain gonadal maturity at 70 mm CL (Vinuesa, 1984), probably at 4 years old. Morphometric maturity is attained in the next molt stage, at 75.4 mm CL (Lovrich et al., 2002b). After the gonadal maturity, males probably continue molting twice a year, in May and October–November, until they are 100 mm CL (Lovrich et al., 2002b). Thereafter, males molt annually mainly in May (106.78 mm CL of adult crabs of present study).

**Line 192 and 199: Should add the definition for enzyme Unit.**

This information was added in M&M section. Page 9, Line 197

**Line 330-333: There are several factors that could effect on the quality of larvae, please consider carefully in your conclusion.**

Thank you for this comment. We agree that there are several factors that could affect the quality of larvae. This point was rewritten in the discussion section. Page 16, Line 354.

**Line 312-333: The author linked the lipase activity in the midgut, lipid content to the ovary maturation, but did not investigate the lipid content in the ovary. Moreover, the developmental stages of ovary/gonad were not described in this study**.

The reviewer is right; we did not investigate the lipid content in the ovary. However, previous studies demonstrated that the females undergo secondary vitellogenesis, accumulation of lipo-proteins in the oocytes, all years between April and December. We did not describe the developmental stages of *L. santolla* ovary since that exceeds the aim of our work. Even though, we added a sentence about this topic. Page 15, Line 341.

**Line 435-438: The data from this study could not support your conclusion**
This point was rewritten in the discussion section. Page 19, Line 459.

**Reviewer #3: This study achieved its objectives to study and compare the biochemical differences and changes of *L. santolla*.**

**In materials and methods section, please justify the use of only 12 juveniles, 10 pre adults and 10 adults crab. Please cite other study or explain if the number of crabs is sufficient for this study.**
We choose this number of animals for different reasons: 1) the sample size was a compromise between analytical ability, precision, and natural variability. 2) The small standard error values obtained in all biochemical determinations. We calculated the variation coefficient of variables and these values were <10%. 3) We estimate the optimum number of samples according to Sokal and Rohlf (1995) equation. This equation takes into account the standard deviation, the mean and the level of precision, and in all cases, the n optimum value obtained was between 4 and 10 crabs.

**The discussion section can be further improved. Suggestions to improve this section are as below:
1. lack of paper cite on feeding behavior, diet and relation to changes in biochemical compounds in the body**

Feeding behaviour and diet information of *L. santolla* were added. Page 15, Line 325. In addition, most works have been performed on the biochemical changes of the body as a response to different diets and not on the natural ontogenetic changes of juvenile or adult wild animals, as it was the objective of this work. In other studies made on larvae of different crustaceans species, the biochemical changes (reserves or enzymes such as proteinase, lipases, LDH, others) was analysed between the different development larval stages. However, since larval development is different from the growth stage of juveniles and adult crabs, we prefer not to discuss our results comparing with changes in larval development.

**2. utilization of fatty acids during crab maturation**

This information was added in Discussion section. Page 15, Line 341.

**3. authors suggested that a/a in muscle reflects the need of that specific amino acid in diets, need to be further elobarate. Essential amino acids is essential. The higher level perhaps contribute by other factors?**

This topic was further elaborated in Discussion section. Essential amino acids are those that cannot be synthesized *de novo* and are only incorporated through diet. Their concentrations within the biomass of crustaceans only vary due to factors such as quality and quantity of food. Page 17, Line 355.

**4. in line 34, authors give support from other species, however it is not being clear whether these animal is also from wild or from cultured thus changes may due to the tailored feed given.**

We understand that the reviewer refers to line 374 of the discussion…..Hence in this version we clarify animal condition (wild or cultured). Page 18, Line 399

Sincerily yours,

Hernán Sacristán
